# Author Correction: Electrochemical Sensor for Detection of miRs Based on the Differential Effect of Competitive Structures in The p19 Function

**DOI:** 10.1038/s41598-018-25536-z

**Published:** 2018-06-04

**Authors:** E. Ghazizadeh, R. K. Oskuee, M. R. Jaafari, S. Hosseinkhani

**Affiliations:** 1 Department of Medical Biotechnology, School of Medicine, Mashhad University of Medical Sciences, Mashhad, Iran; 2 Biotechnology Research Center, Pharmaceutical Technology Institute, Mashhad University of Medical Sciences, Mashhad, Iran; 3 Department of Pharmaceutical Nanotechnology, School of Pharmacy, Mashhad University of Medical Sciences, Mashhad, Iran; 4 Department of Biochemistry, Faculty of Biological Sciences, Tarbiat Modares University, Tehran, Iran

Correction to: *Scientific Reports* 10.1038/s41598-018-22098-y, published online 28 February 2018

The original version of this Article contained an error in Affiliation 2 which was incorrectly given as ‘Biotechnology Research Center, Nanotechnology Research Center, School of Pharmacy, Mashhad University of Medical Sciences, Mashhad, Iran’. The correct affiliation is listed below:

Biotechnology Research Center, Pharmaceutical Technology Institute, Mashhad University of Medical Sciences, Mashhad, Iran.

In addition, the original version of this Article omitted an affiliation for M. R. Jaafari. The correct affiliations are listed below:

Biotechnology Research Center, Pharmaceutical Technology Institute, Mashhad University of Medical Sciences, Mashhad, Iran.

Department of Pharmaceutical Nanotechnology, School of Pharmacy, Mashhad University of Medical Sciences, Mashhad, Iran.

These errors have now been corrected in the HTML and PDF versions of this Article, and in the accompanying Supplementary Information file.

